# **Towards a Less Ideal Theory About Well-being**—**The Case of Post COVID Condition**

**DOI:** 10.1007/s11673-025-10474-z

**Published:** 2025-09-11

**Authors:** Erik Gustavsson, Ericka Johnson, Richard Levi

**Affiliations:** 1 Division of Philosophy and Applied Ethics, Department of Culture and Society, Linköping University, 581 83 Linköping, Sweden; 2 The National Centre for Priorities in Health, Department of Health, Medicine and Caring Sciences, Linköping University, Linköping, Sweden; 3 Division of Gender Studies, Department of Thematic Studies, Linköping University, Linköping, Sweden; 4 Department of Health, Medicine, and Caring Sciences, Division of Prevention, Rehabilitation and Community Medicine, Linköping University, Linköping, Sweden

**Keywords:** Post Covid, Long Covid, Well-being, Rehabilitation medicine, Relational approaches

## Abstract

Post COVID-19 Condition (PCC) is a complex condition presenting significant challenges for patients. Individuals suffering from severe PCC are often assessed in rehabilitation medicine departments or specialized post-COVID centres, where their condition is evaluated using the International Classification of Functioning, Disability and Health (ICF). The ICF framework primarily focuses on functional impairments, disabilities, and restrictions in participation, with an emphasis on the concept of “functioning.” However, a critical question remains: how does this notion of functioning relate to the well-being of these individuals? This paper explores this issue by examining three fictionalized but typical case studies of PCC patients in relation to two distinct theoretical approaches. First, we engage with theories about well-being from the philosophy of well-being emphasizing the individual’s perspective. Second, we explore relational approaches in bioethics and their theoretical underpinnings, which emphasize how people are situated, considering context and relations rather than purely individual conditions. The paper highlights the potential tensions between these approaches while arguing that a more comprehensive understanding of well-being can emerge by integrating insights from both traditions. Through the examination of PCC patient cases, we propose that well-being can be better understood when approached from multiple angles, enriching the understanding of patient outcomes in rehabilitation medicine.

## Introduction

Post COVID-19 Condition (PCC) is a notoriously complex condition posing challenges for many people around the world. The development of PCC is only loosely related to the severity of the preceding infection, and the predominant symptoms, typically including (but not restricted to) disabling fatigue and stimuli hypersensitivity, are difficult to objectivize by laboratory tests or imaging. Nevertheless, many affected patients complain of severely impaired well-being, whereas some patients seem to cope well with their situation. Patients with PCC are often assessed and treated by general practitioners, departments of rehabilitation medicine, or in some instances, by specialized post-COVID centres. Here, the patient’s condition is often evaluated according to the International Classification of Functioning, Disability and Health (ICF). This classification focuses on structural and/or functional impairments, disability, and restriction in participation. Primary importance is ascribed to the concept of “functioning” (WHO 2024). Post Covid-19 Condition is but one example of a disorder characterized by prominently impaired self-reported well-being, fatigue and other ill-defined symptoms in the absence of clear-cut objective findings. Other examples include chronic fatigue syndrome, fibromyalgia, chronic idiopathic pain conditions, and “exhaustion syndrome” (Kleinstäuber et al. 2023). In such situations, the relationship between impaired functioning, primarily or solely dependent on assessments by the patient him/herself, and impaired well-being becomes crucially important. However, the relation between these concepts is rarely discussed from a theoretical point of view in a medical context.

This paper undertakes this task by relating three fictionalized but typical case studies of PCC from rehabilitation medicine to two more theoretical strands of inquiry. First, we outline a theoretical discussion about well-being in philosophy, one which focuses on “how well it is going for the person whose life it is,” an essentially individualistic account of well-being taking individuals as the starting point (see e.g. Fletcher 2013; Lin 2017; Parfit 1984; Sumner 1996). Second, we present approaches in the bioethics literature presenting themselves as “relational,” emphasizing “how a person is situated,” starting their analysis from the relational rather than the individual (see e.g. Gary 2023; Jennings 2019; Koggel et al. 2022).^1^ Whereas the individualistic approach to well-being primarily draws on philosophical work from an analytic tradition, the relational approach primarily draws on work in, among other areas, feminist theory. Somewhat surprisingly, these two strands of theory rarely communicate (Robeyns 2017, 118–119). But representatives from feminist theory may worry that, for example, analytic philosophy abstracts from much real-world complexity in its approach, whereas representatives from analytic philosophy may worry that, for example, the relational perspective focuses too much on contextual factors that have no real relevance for the theoretical question. Within the context of this paper, we engage rehabilitation medicine with these two strands of more theoretical inquiry, as they pertain to the three PCC patient cases. The working hypothesis being that the notion of well-being can be better understood by being approached from multiple directions, engaging elements of different strands of theory.

The paper proceeds as follows. In Sect. 2, we give the reader some tentative characteristics about PCC and describe three fictionalized but typical profiles of patients with PCC. In Sect. 3, we do three things. First, we outline the key theoretical approach in rehabilitative medicine, i.e. the ICF taxonomy. Second, we spell out the individualistic account of well-being drawing on the philosophical discussion on well-being, and third, we outline a relational approach drawing on feminist theory. In Sect. 4, we relate the three cases outlined in Sect. 2 to the theoretical strands described in Sect. 3 and delineate some of the differences with the two approaches by specifying what kinds of questions they would ask—of the person, their experiences, and their social context—when approaching a person with PCC. In Sect. 5, we sum up and conclude.

## Post COVID-19 Condition (PCC) and Three Cases

### Post COVID-19 Condition (PCC)

Post Covid-19 Condition refers to a condition that is medically defined as one which “develops during or after an infection consistent with COVID-19, continue for > 12 weeks, and is not explained by other diagnosis” (NICE 2022, 2). Although this condition is sometimes referred to as “Long COVID” or “Post COVID Syndrome,” we adhere to the terminology used by the World Health Organization and refer to it as PCC. Common symptoms of PCC comprise fatigue, cognitive, affective and/or cardiorespiratory impairment, among others. In many cases, the subjective symptoms are difficult to objectivize. For example, symptoms such as pain, dyspnea, “brain fog,” and fatigue can only be experienced directly from a first-person perspective, and may commonly occur in the absence of any corresponding pathology found in terms of clinical signs or findings in laboratory and imaging assessments. The recent pandemic has created a global scientific as well as public focus on the disorder, where major controversies are played out even as regards accepting the existence of the condition as such. Among sceptics, the report from patients that they are badly off in terms of well-being due to COVID-19 is questioned as a sufficient ground for labelling PCC a bona fide *medical* diagnosis (rather than, for example, a psychiatric or “cultural” disorder). From this perspective, the “mere” subjective assessment of the patient regarding impaired well-being demands corroboration by the third person perspective in “objective” terms (e.g. pathological physiological parameters) in order to count. Thus, by a sceptical account, *uncorroborated* fatigue, pain, “brain fog,” anhedonia *et cetera* is considered insufficient as reasons for accepting subjective reports of impaired well-being as a matter of medical concern.

There is a growing body of empirical data on how people are affected by PCC (see e.g. Sigfrid et al. 2021; Pearson et al. 2022). To assess the extent to which PCC may impact well-being and how effective various measures are in targeting the condition in this respect, a variety of instruments are being used, such as EQ-5D, SF-36, etc. These outcome measures all have some implicit notion of what is good (and bad) for these patients, however, such notions, are usually not made explicit.

Although PCC is a complex condition with a plethora of possible symptoms, we shall, in the following, outline three “prototypical” cases in order to enable the subsequent discussion. In describing these profiles, we draw on a large number of research studies hitherto published on characteristics of lingering symptoms and signs in persons with PCC, including empirical data from studies, comprising a retro- and prospectively investigated cohort of over four hundred persons over a period of two years after hospital-treated SARS-COVID-19 infection (Wahlgren et al. 2023). Although the presented cases have been fictionalized as to ensure anonymity, they do reflect the diversity seen in terms of variation in types and degrees of experienced symptoms as well as in terms of reported impact of persisting impairments as it pertains to well-being.

### Three Different Profiles of Cases With PCC

#### The Physician

Forty-two-year-old female physician, mother of three young children. Non-smoker, very moderate drinker. Happily married with husband with high workload in qualified job. Previously healthy, with exception of one minor depressive episode in late teens. Prior to COVID-19, worked full-time as hospital physician including regular on-call duties. In spare time avid exerciser at least four times/week. Has completed several Marathons. Fell ill with COVID-19 April 2020, not hospitalized, typical flu-like symptoms for ten days. Returned to full-time work two weeks later. Experienced severe “brain fog,” fatigue, stress intolerance, episodes of pain in chest and muscles. Went on full-time sick leave for over one year, commenced outpatient rehabilitation. Persisting problems in coping with taking care of the children. Emotionally labile. Exercise intolerance. Severe chronic fatigue and sleep disturbance. Lab tests normal. Neuropsychological testing with average (normal) results. Now manages to work 25 per cent of full-time with customized tasks, no on-call duties. Can ride a bike for 5 km at a calm pace each day, but not without some post-exertional malaise. Feels that she is “not herself” anymore, feels low, but not clinically depressed. Describes herself as “a shadow of my former self.”

#### The Carpenter

Fifty-six-year-old male carpenter. Two children, grown up and out of the house. Ex-smoker, quit eight years ago. Heavy beer drinker, but not alcoholic. Married with wife working as assistant nurse. Premorbid obesity, hypertension, type 2 diabetes mellitus, cervical spine arthrosis. Nevertheless, prior to COVID-19, worked full-time, but contemplated partial pension due to neck problems. In spare time socially very active member in motorcycle club. Fell ill with COVID-19 late March 2020, hospitalized with severe respiratory problems, treated by ventilator in intensive care unit for over a month, post-acute inpatient rehab due to critical illness polyneuromyopathy (CIP/CIM). Discharged home after two months in hospital. Persisting problems with exertional dyspnea, increased physical and psychological fatigability, these symptoms also objectivized by testing. Decided to retire full-time in fall of 2020. Has restricted his social life to some extent but still enjoys weekly motorcycle excursions with the club. Reports good life satisfaction, claims to have coped well in his new situation, feels happy to have survived the infection with moderate limitations and feels no need for any intervention.

#### The Shop Assistant

Thirty-four-year-old female shop assistant, single, no children. Smokes fifteen cigarettes/day. “Average” alcohol consumption. Premorbid obesity, fibromyalgia, recurrent depressive episodes, generalized anxiety disorder. Prior to COVID-19, worked full-time but with recurrent short-term sick leave periods. Fell ill with COVID-19 in July 2020, not hospitalized, typical flu-like symptoms for a week. Persisting multiple symptomatology, including headache, chest pain, muscle aches, increased anxiety and listlessness, fatigue, post-exertional malaise, sleep disturbance, fleeting sensations of pins and needles across the body. No significant aberrations in objective lab tests or physiological tests. Been on full-time sick leave since initial illness. Reports very poor life satisfaction. No positive response to several periods of outpatient rehabilitation programmes during the last two years.

As these cases illustrate, the premorbid clinical status, objective initial disease severity and the objective versus subjective assessment of the post- COVID-19 situation varies substantially. The consequences in terms of impact on well-being may clearly not be proportional to either initial disease severity or measures of objective functioning.

## Three Perspectives on Well-being

### Well-being in the Context of Rehabilitation Medicine: The ICF Taxonomy

Rehabilitation medicine is a sub-specialty specifically focused on the *consequences* of medical conditions in terms of daily living. It aims at minimizing disability and optimizing functioning according to a biopsychosocial paradigm. From a theoretical point of view, and in accordance with modern medicine in general, it utilizes the ICD (International Classification of Diseases) for diagnosing the *etiology* of a condition, and the ICF (International Classification of Functioning, Disability and Health) for classification of the *consequences* of a condition. ICF is of primary importance in Rehabilitation medicine as well as in other medical branches dealing with rehabilitative efforts and is currently considered the taxonomy most pertinent for both classification and for clinical practice.

**Fig. 1:**
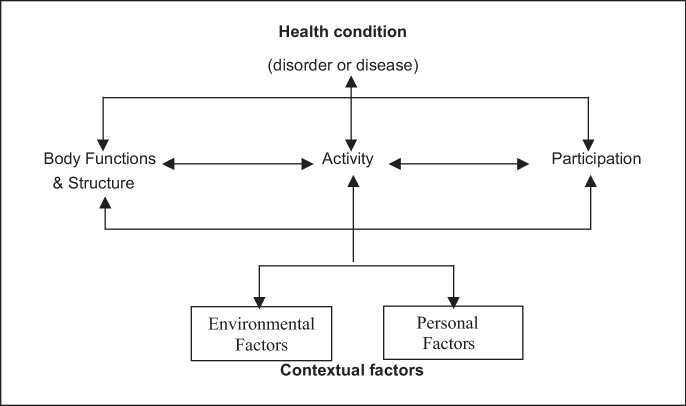
ICF model

International Classification of Functioning comprises a classification of health and health-related domains. It is the WHO framework for measuring health and disability at both individual and population levels. International Classification of Functioning is well-developed for broadly assessing “functioning”, which according to its taxonomy is separated into “body structures and body functions,” “activities” and “participation.” In addition, it promotes (but does not explicitly structure) assessment of environmental as well as personal factors (Fig. 1) (WHO 2002).

The ICF does not specifically include measures of well-being. However, both in clinical practice and in medical research, it is nowadays acknowledged as a matter of course that assessment of overall well-being also is required, typically in the guise of “quality of life” and/or “life satisfaction.” Therefore, ICF is sometimes complemented by instruments designed to specifically assess these issues, e.g. by the WHO-5 Well- Being Index or, more commonly, EQ-5D, the latter of which assesses health-related quality of life in terms of five dimensions: mobility, self-care, usual activities, pain/discomfort, and anxiety/depression.

However, the very concept of well-being is rarely discussed from a theoretical point of view in the medical context. In contrast, the assumption seems to be that attainment of maximal “functioning” (as measured by ICF) will also lead to the attainment of the maximally achievable well-being in light of the extant disorder or disability.

It may thus broadly be stated that rehabilitative medicine focuses on the direct pathophysiological consequences of a disease or injury as they translate into impairments of functioning. It tends to accept as well-being what EQ-5D or similar indices tacitly or explicitly comprise. However, in order to ensure that such instruments really measure what is taken to be important, it seems fundamental to first scrutinize how the concept of well-being is best understood. This seems to be a question for the philosophy of well-being. However, it seems unlikely that a person’s well-being is constituted in splendid isolation from contextual factors, e.g. cultural norms, socio-economic factors, etcetera. Thus, the situatedness of the individual in such terms also needs to be considered. But let us begin with the philosophy of well-being.

### The Philosophy of Well-being

There are many different, often conflicting, ideas about what makes a life go well. These different ideas may have quite different implications for how different kinds of medical disorders and treatments are being understood and handled in clinical practice as well as in healthcare policy. For example, they are crucial in assessments of how severe a given disease is considered to be and in predictions about the efficacy of healthcare interventions which, in turn, often constitute criteria for resource allocation in healthcare systems (see e.g. Barra et al. 2020). In some countries, it may also influence decisions on, for example, rights to sick leave and provision of rehabilitative measures at the expense of society (Carr et al. 2001).

In the following, we focus on accounts of well-being rather than theories about illness or health.^2^ We proceed accordingly because we are interested in the evaluative aspects of people’s lives, and for theories about illness and health to account for such aspects, they would, in the end, have to refer to more fundamental views about what make people’s lives go well—views about well-being. The tradition of moral philosophy offers several ways in which theories on well-being can be divided (Fletcher 2013; Kagan 1992; Parfit 1984). The standard way of dividing among theories on well-being in this tradition stems from an appendix in Derek Parfit’s book *Reasons and Persons* published in 1984. In this tradition, well-being refers to “a life going well for the person whose life it is” (see e.g. Sumner 1996, 20),^3^ or “what is ultimately good for a person” (Crisp 2021).

Parfit (1984, 493–502) distinguished between three kinds of theories: hedonistic theories, desire-fulfilment theories, and objective list theories. *Hedonistic theories* according to which a life goes well if it contains a balance of pleasure over pain. The hedonistic thesis is that experiences of pleasure and pain are the only relevant elements for a person’s well-being. *Desire-fulfilment theories* according to which a life goes well if one gets what one wants, prefers, or desires. A person’s well-being is thus constituted by a balance of desire-fulfilment over having one’s desires frustrated or having one’s aversions fulfilled. *Objective list theories* according to which certain things make a life go well, and these things are considered to do so whether a person wants to have these things or not. Things that usually appear on such lists are freedom, love, pleasure, and virtue.

Distinguishing between objective and subjective theories is one of the crucial manoeuvres here. While this distinction may seem quite straightforward, its importance for theories about well-being, and consequently for healthcare practices, calls for a closer examination. Although Parfit characterized objective list theories as being objective in the sense that”…certain things are good or bad for us, whether or not we want to have the good things, or to avoid the bad things” (Parfit 1984, 493), the standard interpretation of these theories is that the good things are objectively good in the sense that they are considered to be so, irrespective of the person’s attitude towards them. For example, it is good for you to be free irrespective of whether you want to be free or not. According to this interpretation it may be claimed that hedonism is objective in the same sense as objective list theories are (Crisp 2021; Fletcher 2013), given that one can experience pleasure that one does not want to experience. That is, one may experience pleasure meanwhile one does not have a favourable attitude towards that pleasure. This is the sense in which hedonism may be interpreted as an objective list theory (but with only one thing on its list). Perhaps guilty pleasures are such examples, you may experience pleasure while torturing cats but you wish you did not. Accordingly, the main idea is that these theories are subjective in the sense that they are dependent on the individual’s attitudes. For example, Lin (2017) states that “ … subjectivism says that something is basically good for you just if, and to the extent that, you have a certain favorable attitude, *A*, toward it under the right conditions, *C*” (354).^4^ Accordingly, the defining characteristic for the subjective/objective distinction is attitude-dependence. This leaves us with desire-fulfilment theories as the only example of subjective theories about well-being.

Let us pause here and take a step back. These accounts about well-being may strike one as too abstract. Indeed, there is a general worry within analytic philosophy that the level of abstractness and idealization at which analytic philosophy is taking place make theories unable to account for the messiness of everyday life (Burman 2023). The more ideal a theory is, the more it disregards the complexities in everyday life. By abstracting aspects away (as is necessary in ideal theorizing) one risks make important aspects invisible.

*The carpenter, the physician,* and *the shop-assistant* are embedded in various kinds of relations. Consider, for example, a hedonistic evaluation of *the physician’s* well-being. According to such an evaluation, her relations to her children and husband are relevant to the extent they contribute to her experienced pleasure. Accordingly, her relations may well be instrumentally valuable but do not have any value for their own sake. Consider next, objective-list theories, here some relations may be characterized as objective goods. For example, Fletcher (2013) suggests that friendship is one such good. Accordingly, objective list-theories may account for the importance of relations as constituents of well-being. 

How would this issue be understood by adherents of relational approaches? One may suggest that the correct answer here would be that they typically would adhere to objective list theories about well-being. However, relational approaches want to do something quite different. In the next section we spell out our interpretation of the relevance of their project for understanding the notion of well-being.

### The Individual, the Collective, and the Relational

Hitherto, we have discussed individualistic accounts of well-being. In “What is Group Well-being?,” Wiland (2022) discusses the difficulties and importance of thinking about the relationship between an individual’s well-being and the well-being of a group. He pedagogically shows how measuring this is much more complicated than understanding a group’s well-being in terms of the addition of the well-being of the individuals making up the group or an average of the same individuals’ level of well-being. Rather we must think about how the well-being of individuals and groups affects each other, in top-down and bottom-up ways and the external factors that may be simultaneously affecting them both. What his arguments point to, among other things, is that groups come in many different shapes, forms, and *raisons d’etre*. What the group looks like, how it works, and what it does, matters for the question of how an individual fares in it. If we are interested in a person’s well-being as related to the group’s well-being, we need to have tools to understand the relationship between the individual and the group as well as the relations between different members in the group.

Tools to figure out that relationship do, however, exist. In the literature on bioethics, there is an increase in approaches that refer to themselves as “relational.” However, relational approaches may be relational in several different ways. Gary (2023) spells out four kinds of difference in this respect. In her own words: “I emphasize four axes of difference rather than distinct types of relational approaches in order to more closely capture how they appear in the literature …” (Gary 2023, 734). Whereas the relational accounts referenced by Gary have primarily focused on questions about relational autonomy and care ethics as well as the relation between humans and non-human things, we are interested in how a relational angle on the notion of well-being may play out.

The first difference has to do with* the scope of relations considered* which is about which beings that can constitute a part of the relevant relation. For example, while some approaches ascribe responsibilities to certain relations between human beings, other would include relations to non-human animals as well as inanimate things.

The second difference concerns *the nature of relations considered,* more specifically, the *kinds* of relations that are considered primary for theorizing. Gary contrasts between, on the one hand, accounts that primarily focus on structural relations and, on the other hand, those that focus on more-than-human relations. The former kind of approach focuses on structural relations rather than interpersonal relations since these relations are crucial for understanding how people situate themselves. For example, in the words of Koggel “ … those who are oppressed are forced to situate themselves in relation to their oppressors” (Koggel 1997, 158). The latter approaches focus on more-than-human relations which may involve everything from human and non‐human animals to ecosystems and plant species to technological and social artifacts.

The third difference is about *causal versus constitutive accounts of selfhood* which is concerned with to what extent an account takes relations to be determinative of selfhood. According to causal accounts, relations are necessary for developing certain capacities (such as reflection and deliberation), whereas constitutive views say something more, namely that “ … relationships are ontologically prior to individuals …” (Gary 2023, 737, see also Mackenzie and Stoljar 2000, 22).

The fourth difference is about *integrity of individual selfhood*. This difference is related to the causal versus constitutive difference. That is, if one accepts that relations are prior to individuals, to what extent is there an individual self left to discuss?

Furthermore, sometimes there are relational aspects which become (in)visible because of the methodological approaches employed. While this is not the place for an extended discussion on theories of science, the interdisciplinary approach of this paper and the larger meta-epistemology project we are part of has brought together authors with three very different academic backgrounds working in radically different knowledge paradigms. One aspect of collaborating across disciplines and paradigms that we continuously are dealing with is trying to make sense of the “same” research object (in our case, PCC) when our research methods are producing very different understandings of that object. But even within the same disciplinary areas, methodological diversity creates ontological complexity. For example, some more traditional methods within sociology include ethnographic observations, surveys, group or individual interviews, but these have been complemented by more recent ones which rely on computer analysis of demographic data, data sometimes gathered by states, other times by private actors, and sometimes harvested out of the internet. Sometimes this methodological and ontological complexity is very frustrating. But it can also be useful. For example, qualitative methodological tools like observation and interviews can produce materials which help us see and articulate the group/individual relationship and its implications for health. Not only does this complement more traditional quantitative measures of health that form the basis of many PCC conversations, but such qualitative methodological work when connected to critical theory may shift a conversation about, for example, *causal vs constitutive* to a conversation about an individual’s relation to social expectations, power structures and the distribution of responsibility for oneself and others across multiple actors (individuals—partners, children, care providers and groups—families, work places, congregations, healthcare structures, etc.).

Back in the 1950’s, Parsons looked at this in his theorizing about the sick role and how a person’s health status was legitimated through different roles they had within a group and how this was related to the groups need for individuals to be healthy. In another mid-century American work, this time on mental health in rural areas, Eaton and Weil spoke about the individual and group by exploring different ways groups take care of (and sometimes repress) individuals (Eaton and Weil 1955). A bit later, and a bit more radically, Illich (1975) criticized the way modern medicine created a fissure between the individual from their group, leading to less well-being for both. Individuals which did not have the group to take care of them were isolated and less well-off. At the same time, groups with individuals who were not well integrated did not function as well. One of his many points was that as modern medicine took over the role of healthcare, “care” became less of a concern for the family, which isolated modern individuals and simultaneously destroyed much of what was good about the family group.

The field of women’s health has also often touched on the complex relationships between individuals and the expectations different groups (families, professions, social collectives of various sorts) make upon women. In this research, it becomes very clear that the well-being of a group can increase in an inverse relationship to the individual’s well-being if that individual is expected to provide work and care for others in the group in disproportionate and exploitative ways (see Davis 2002; Martin 1987; Rapp 1998; Thompson 2005; Cacchioni 2015).

Gender is not the only line along which power dynamics travel that has significance for health in the relationship between a group and individual. Theories about power and age, class, race, educational level, sexuality, gender identity, geographical location, legal regulations, etc., all show their analytical significance if we want to discuss the well-being of individuals and groups. There is a large body of work on this coming from fields like critical medical humanities (Whitehead and Woods 2016; Wailoo et al. 2010; Williams et al. 2011), exploring how power dynamics, group health and individual health are related to well-being. Tangential to this work is also the research that shows how material constraints and affordances of medical technologies play together to produce bodies, diseases, and health (Mol 2002; Löwy 2015). This work has fed into studies from within feminist technoscience and STS that is concerned with the messy entanglements producing material-discursive subjectivities in situated contexts. Other approaches, for example within feminist phenomenology, are more focused on the subject’s lived experiences that create an understanding of the self (Käll and Zeiler forthcoming). But all of these are interested in relational ways of being in and with the world and how this shapes, affords, impacts, or allows for the experience of the subject.

Similar methods and insights have also already been applied specifically to PCC. For example, in the afterword of *Under the Skin*, Villarosa uses her analysis of the construction of black health to examine how black Americans have been impacted by the COVID-19 pandemic. There are surely soon many more to come. In the framework of our discussion, we can take inspiration from these approaches to see how collectives and their responsibilities for and expectations on individuals may be impacting the three patients described below. This approach will then make relevant different potential causes or constituents of well-being, with a sensitivity to the contextual power structures within which they are living.

## Discussion—Tensions and Possibilities

Rehabilitation medicine, the philosophy of well-being, and relational approaches will highlight different parts of the lives of *the physician*, *the carpenter*, and *the shop-assistant*. However, not only do the different perspectives focus on different features of these lives, but there are also tensions between these perspectives that arise when trying to make sense of the three cases, more specifically. In the following, we shall focus our discussion on three themes: the challenges of upholding the subjectivity/objectivity distinction, how relations are best accounted for, and the role of premorbidity and expectations. The discussion hitherto suggests that an exploration of these different strands of theory in relation to well-being may open for new ways in which theoretical discussions about well-being can make sense of complex conditions such as PCC.

### Challenges with upholding the subjective/objective distinction

From a philosophy of well-being perspective, the subjective/objective question at stake is whether something can contribute to a person’s well-being irrespective of that person’s attitudes towards that something. That is, according to objectivism it follows that someone else, such as a physician or a friend, may know better how well off you are than you do. Such views open for the possibility that you can be wrong about your level of well-being. For example, drawing on the objective list theory proposed by Fletcher (2013) according to which “achievement” and “friendship” are examples of items that make your life go well irrespective of the extent to which you care about these things.

Compare next *the physician* and *the carpenter*. Whereas *the physician* has a persisting neuropsychological and physical ability within normal limits in comparison with an average person, *the carpenter* had more distinct persisting limitations. That is, according to a theory of well-being with an objective flavour *the carpenter* seems to be worse off than *the physician*. However, while *the carpenter* reports a good subjective outcome and feels no need for further healthcare or social interventions at follow-up, *the physician* felt devastated by her new situation. Accordingly, *the carpenter* is better off along the subjective dimensions of well-being, whereas *the physician* is better off along the objective dimensions. A putative reaction from the philosophy of well-being camp here would be the following: if this conclusion about these two individuals is correct, one still wonders which dimension matters more. Which of these two dimensions of well-being is more important?

However, the strict distinction between the subjective and the objective in the philosophy of well-being presupposes an individual person as its starting point. But a relational approach would problematize this. For example, consider the difference between causal and constitutive relations discussed by Gary (2023). To the extent that relations are considered to be constitutive rather than causal the subjective/objective distinction is difficult to uphold. As soon as we engage methods which make visible the wider relational context within which a person acts (and is acted upon)—or is entangled within—we are able to see the structural and contextual aspects of well-being. The worry here would be that too abstract accounts of well-being would not only disregard these issues but also contribute to making them invisible. This also raises the question of how the relation between, on the one hand, the subjective/objective distinction, and on the other hand, the individual/collective distinction should be understood. Listening to the subjective and listening to the individual in relation to the collective could both be interpreted as engaging an epistemological critique against the “expertise” of the medical and against the assumed appropriate and natural position of individuals in collective relations. But a relational approach would direct analytical attention to the power dynamics that produce an individual’s ability to claim knowledge about our well-being. It would also direct analytical attention to the relative privilege of some subjects' positions in relation to others.

### Accounting for Relations

One may want to consider the people relying on the patient for their daily needs, as well as how many other people are serving the other person’s daily needs. For example, considering the relational care expected from and provided to patients would bring into view different possible caring expectations put on the individuals; *the physician* has shared responsibility for providing care for small children. Neither *the carpenter* nor *the shop assistant* does. While these relations would be accounted for in the individualistic accounts of well-being, their role would be peripheral. Since individual’s well-being is probably intimately tied to the well-being of those whom they are responsible for, this may be a shortcoming of these theories of well-being. Likewise, *the carpenter* is married to an assistant nurse. This may give him a very different level of care at home than that (not) provided to *the shop assistant* and possibly affect his lived experience of well-being.

Another element of the collective’s impact on well-being would be made visible through analysis of the structural power dynamics that the individuals encounter in their existence within a neoliberal capitalist state, albeit the one based on a concept of welfare as found in, for example, Sweden. Both *the physician* and *the shop assistant* are still “expected” to engage in productive work outside the home and for a salary. To legitimately not do this, they would need to convince a series of gate-keepers (from medical doctors to social security bureaucrats approving sick-leave) that they are, indeed, too ill to work full time and thus are deserving of paid sick-leave. This is a very different situation than that which *the carpenter* is in, who has retired and will receive his pension regardless of how sick he is considered by the medical community and social services. These three patients then have very different stakes in being diagnosed as ill.

The above studies mentioned at the beginning of this section are primarily concerned about the well-being of individuals and groups, but they engage methodological tools that make visible the other, external, structural, often related to power, issues that impact how an individual is positioned (what subjectivity they are allowed to have) by the dynamics internal and external to the group. In some ways, this posits that well-being is distributed—or being produced and reproduced in continuous fluxes of care practices, imaginaries, norms, expectations. We suggest that an interdisciplinary approach to understanding well-being that opens for methodological tools that make visible power beyond the agency assumed to be accrued in the body of an individual would allow more structural and relational elements of well-being to be explored. This would let us discuss well-being in relation to others, and well-being in relation to the expectations and affordances society makes on individuals.

Wiland’s paper broaches these questions, even if he doesn’t use those words. However, to make this epistemologically relevant to the conversations within a meta-epistemological framework, we suggest that it is important to recognize that there exist methods that can be used to explore the relational health dynamics between individuals, groups, and external structures. There are people who have found ways to explore how care standards and social norms frame the imaginaries of health available to patients (Mol and Berg 1998), for example. There have been studies that look at how legal definitions of care provision are entangled with diagnostic measures of disease severity (Johnson et al. 2016). And there are activists and researchers who have jumped into the “belly of the beast” and produced research that simultaneously tries to change the diagnostic and treatment opportunities available to patients (BWHBC 1973; Murphy 2012; Zuiderent-Jerek 2016).

Wiland is right, how those dynamics play out is contingent on many different things. The next step is to try to figure out what those things are. Luckily, we don’t have to re-invent the wheel to do that. That is one contribution the medical humanities and the social sciences can bring to a meta-epistemological study of PCC.

### The Role of Premorbidity and Expectations

How should one understand the mismatch between subjective and objective dimensions? It may be hypothesized that *the carpenter* was able to cope much better with necessary adjustments due to a very different life situation as compared to *the physician*. Also, *the physician* most likely had a premorbid intellectual and physical ability substantially superior to average and expected herself to perform at a very high level.

It may also be hypothesized that both *the physician* and *the shop assistant* manifest a vulnerability to PCC, albeit for very different reasons: *the shop assistant* clearly displayed a premorbid hypersensitivity to average life stressors, whereas *the physician* displayed very high premorbid claims on her capacity, which she used to the limit of her (excellent) capability, thus leaving no slack for any degree of added limitations. The notion of premorbidity plays an important role in rehabilitation medicine. “Premorbid,” in this context, refers to the health status of the person before they fell sick with COVID-19/PCC. Consequently, from the perspective of rehabilitation medicine, it refers to the level of function a person had before they got COVID-19. This notion also relates to the role that aspirations have in that they determine the reference point for intrapersonal comparations of well-being from a subjective perspective. From the patient’s perspective, it may appear obvious that well-being is understood in relation to their previous experiences of a good life. This, in turn, relates to what aspirations the person had on life before COVID-19 and that it is based on those demands that the person estimates their well-being with PCC. In turn, this means that the person’s well-being with PCC is partly determined by what demands the person had on life before PCC.

From this point of view the old adage, “it’s not how you have it but how you take it that matters” seems to play an important part in the ability to cope with PCC, and “how you take it” putatively is influenced by a plethora of factors, including (but not restricted to) current demands in daily life, expectations of oneself, and premobid vulnerability in terms of either low resistance to stressors or extreme expectations of one’s own ability. However, *the physician’s* aspirations on her life is one thing, whereas others’ aspirations on her life is quite another. This relates to the structural relations. Structures produce expectations which impact our aspirations—whether these are the expectations social institutions place on us, or the rules and regulations (articulated and unspoken) an organization or workplace have for us, or the family care dynamics connected to our individual gendered, classed, aged subjectivities, these structural frames create understandings of quality and success that can influence our individual aspirations. These entangled power dynamics also come into play when a patient understands their health.

## Well-being as a Relational or Functional State?

The overarching goal of rehabilitative medicine is to maximize functioning relative to the degree of extant impairments. Well-being (however understood) is, at least tacitly, thought to improve as functioning thereby improves. In cases where a significant mismatch between the patient’s ability to function (as perceived by healthcare professionals) and the patient’s reported well-being remains; this is typically interpreted as a case for psychiatric referral or possibly reflecting either malingering or socio-economic or even existential issues outside the scope of medicine. This raises a number of questions: i) “To what extent is functioning relevant to well-being?”, ii) “To what extent is well-being (or even functioning) a relevant outcome measure in (rehabilitation) medicine?”, and iii) “How should the concept of well-being best understood in a medical context?”.

From the perspective of the philosophy of well-being the question arising would be about the value of functioning. More specifically, if functioning is valuable for its own sake (and thereby a plausible item on an objective list theory) or if functioning has an instrumental value in as so far as it, for example, is conducive to pleasure over pain (and thereby, depending on the extent to which this instrumental value can be established, a relevant consideration for hedonistic theories). As question (iii) indicates, the best account of well-being (in general) may be different from the best account of well-being *relevant for a medical context*. Or at least, it is not obvious which constituents of well-being medicine should be concerned with. There may be constituents of a person’s well-being that medicine should just not be concerned with.

From a relational perspective on well-being, functioning would be considered relevant to well-being in as much as the individual is able to be perceived as functioning within the relations that they are expected to engage in. This well-being is then an outcome of both efforts to address the individual’s health from (rehabilitation) medicine but also efforts to change or adjust the expectations and aspirations upon the individual from social institutions, the organizations and contexts the person engages with, and the dynamics of groups they find themselves part of in daily life. This concept of well-being would have to expand to include not only a medical context, but also the context of the person’s entangled social self.

A satisfactory account of well-being relevant for medicine should not only be able to account for what makes a life go well but also the messiness of the contextual factors, which seems especially important in order to understand complex conditions such as PCC.

## Footnotes

1 While these authors primarily focus on relational approaches to, for example, the notion of autonomy, we shall draw on similar ideas to better understand the notion of well-being.

2 See for example Boorse 1977 and Nordenfelt 1995 for an elaborated discussion about such theories.

3 A terminological note: whereas we use “well-being” to refer to what is ultimately good for a person, others use closely related terms for that purpose such as welfare or prudential value. This is not a substantive difference but mere difference in terminology.

4 For a more elaborated discussion on the notion of attitude-dependence see Fletcher (2013).
